# Major Increase in Incidence of Pediatric ACL Reconstructions From 2005 to 2021: A Study From the Norwegian Knee Ligament Register

**DOI:** 10.1177/03635465231185742

**Published:** 2023-07-27

**Authors:** Caroline E. v. W. Kooy, Rune B. Jakobsen, Anne M. Fenstad, Andreas Persson, Håvard Visnes, Lars Engebretsen, Guri R. Ekås

**Affiliations:** † Oslo Sports Trauma Research, Norwegian School of Sports Science, Oslo, Norway; ‡ Faculty of Medicine, University of Oslo, Oslo, Norway; § Department of Orthopedic Surgery, Akershus University Hospital, Oslo, Norway; ‖ Department of Health Management and Health Economics, Institute of Health and Society, University of Oslo, Oslo, Norway; ¶ The Norwegian Knee Ligament Register, Department of Orthopedic Surgery, Haukeland University Hospital, Bergen, Norway; # Department of Orthopaedic Surgery, Oslo University Hospital Ullevål, Oslo, Norway; ** Department of Orthopedic Surgery, Sorlandet Hospital Kristiansand, Kristiansand, Norway; Investigation performed at Akershus University Hospital and Oslo Sports Trauma Research Center, Norwegian School of Sports Sciences, Oslo, Norway

**Keywords:** pediatric, ACL reconstruction, ACL injury, incidence, register

## Abstract

**Background:**

The incidence of pediatric and adolescent anterior cruciate ligament reconstruction (ACLR) is increasing in several countries. It is uncertain whether this trend applies to countries that traditionally prefer an initial nonoperative treatment approach whenever possible, like Norway. Nationwide, long-term patient-reported outcomes and revision rates after ACLR in the pediatric population are also lacking.

**Purpose:**

To determine the incidence of pediatric ACLR in Norway since 2005, as well as to detect trends in surgical details and describe patient-reported outcomes up to 10 years after ACLR.

**Study Design:**

Descriptive cohort study.

**Methods:**

This study is based on prospectively collected data on girls ≤14 years and boys ≤16 years, registered in the Norwegian Knee Ligament Register at the time of their primary ACLR, between 2005 and 2021. The main outcome was the incidence of ACLR, adjusted to the corresponding population numbers for each year. The time trend was analyzed by comparing the mean of the first and last 3-year period (2005-2007 and 2019-2021). Patient-reported outcomes were assessed using the Knee injury and Osteoarthritis Outcome Score preoperatively and at 2, 5, and 10 years postoperatively.

**Results:**

A total of 1476 patients (1484 cases) were included, with a mean follow-up of 8.1 years (range, 1-17). The incidence of pediatric ACLRs per 100,000 population increased from 18 to 26, which corresponds to an increase of 40% for boys and 55% for girls. Concurrent meniscal procedures increased significantly from 45% to 62%, and the proportion of meniscal repairs increased from 19% to 43% when comparing the first and last time period. The mean Knee injury and Osteoarthritis Outcome Score values for the Sport and Recreation and Quality of Life subscales were between 72 and 75 at the 2-, 5- and 10-year follow-up. The 5-year revision rate was 9.9%.

**Conclusion:**

There was a major increase in incidence of pediatric ACLR in Norway during the study period. There was a shift in the approach to concomitant meniscal procedures from resection to repair, with more than a doubling of the proportion of meniscal repairs. Patient-reported outcomes revealed long-lasting reduced knee function.

An anterior cruciate ligament (ACL) injury is a major injury that severely affects a child’s knee function and ability to participate in daily activities and sports. There is a lack of agreement on what is considered the optimal treatment algorithm for ACL injuries in the pediatric population.^2^ The 2 treatment options, rehabilitation alone and ACL reconstruction (ACLR), both involve risk of complications. Nonoperative treatment may lead to instability and secondary injuries such as meniscal and cartilage damage.^1,2,8,19,36^ Operative treatment involves demanding techniques with risk of growth disturbances and insufficient graft development.^3,4,15,25^ Adherence to activity restrictions during rehabilitation, and the necessary delay in return to sports to prevent new injuries, is often challenging for young children. It may also affect their self-perception and sense of belonging.^28^ Management therefore becomes a balancing act between avoiding reinjury and maintaining an acceptable activity level and knee function. Regardless of treatment, an ACL injury leads to early-onset pain and functional impairment, with increased risk of reinjury.^6,53^ This may culminate in early cartilage degeneration and osteoarthritis, resulting in a reduced quality of life already at a very young age.^14,22-24,49^ The reported increase in incidence of both ACL injuries and ACLRs in the youngest population in several countries recently is therefore of major concern.^7,42,52,55^

As opposed to the United States, where initial ACLR is the preferred treatment algorithm, exercise therapy is usually the first step in most surgical sites in Scandinavia, including Norway.^12,13^ Early ACLR is considered if additional injuries warrant immediate or subacute repair. Delayed ACLR is an option for those who sustain secondary injuries, have recurrent instability, or have unacceptable activity limitations.^11,30^ Given the Norwegian traditional preference for a nonoperative treatment approach, it is uncertain whether the observed increase in ACLRs is also present in the pediatric and adolescent population in Norway.^31^ Trends over time in ACLR incidence and patient characteristics in the pediatric and adolescent population have not been described in Norway or in countries that follow a similar treatment algorithm. In addition, long-term results after ACLR in this population are lacking.

Our primary aim was to determine the annual incidence of pediatric ACLR in Norway over the past 2 decades. Our secondary aims were to describe (1) patient characteristics, (2) trends regarding surgical details including revision surgery, and (3) patient-reported outcomes. Our hypothesis was that the incidence of ACLR in the pediatric population in Norway increased during the study period.

## Methods

### Design and Setting

We conducted this study in accordance with the Strengthening the Reporting of Observational Studies in Epidemiology checklist.^50^ The study is a retrospective analysis of prospectively collected data from the Norwegian Knee Ligament Register (NKLR). Since 2004, this register has collected information on all cases of primary and revision ACLR from both public and private hospitals in Norway.^16^ It contains high-validity data with 86% coverage for primary ACLR, ranging between 77% and 97% in regular coverage analyses from its inception.^16,29,46^ Surgeons report surgical details and intraoperative findings to the register immediately after surgery. The patient-reported outcome is assessed using the Knee injury and Osteoarthritis Outcome Score (KOOS), with 5 subscales: Pain, Symptoms, Quality of Life (QoL), Sport and Recreation (Sport/Rec), and Activities of Daily Living.^21,38^ This self-report questionnaire is completed by patients preoperatively and at 2-, 5-, and 10-year after ACLR. The patient-reported KOOS at the 2-year follow-up has a mean compliance rate of 60% for all ages, ranging from 53% to 69% (2006-2020).^46^

### Study Population

**Figure 1.**
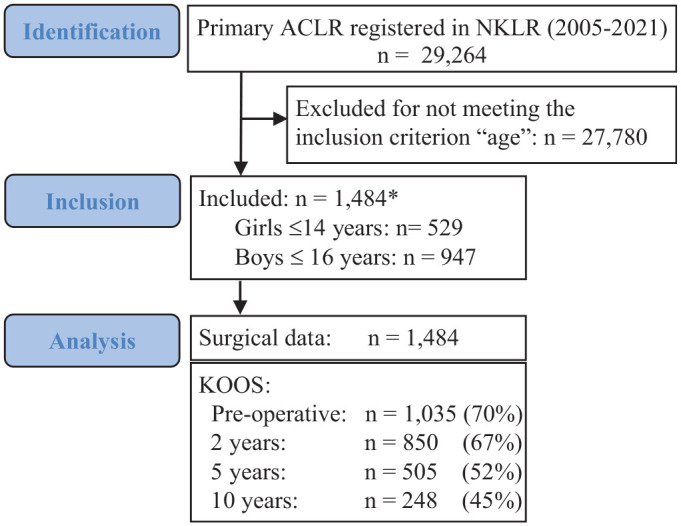
Study flowchart. Asterisk (*) denotes the number of primary anterior cruciate ligament reconstruction (ACLR) cases. Out of 1484 ACLR cases, 8 were bilateral, resulting in a total of 1476 patients with ACLR. The KOOS response rate is presented with the number of responses received. The percentages reflect the proportion of patient responses relative to the total number of patients who had reached sufficient follow-up time for that specific time point. NKLR, Norwegian Knee Ligament Register; KOOS, Knee injury and Osteoarthritis Outcome Score.

All patients registered with primary ACLR in the NKLR between January 1, 2005, and December 31, 2021, were eligible, and girls ≤14 years old and boys ≤16 years old were included (Figure 1). These age cutoffs were set to target the skeletally immature pediatric and adolescent population. Inclusion until 2021 ensured a minimum 1-year follow-up.

### Variables and Outcome Measures

The following variables were included: age, sex, height (cm), weight (kg), activity at the time of injury, date of injury, date of primary and potential revision ACLR, graft choice (hamstring, patellar, and quadriceps tendon autografts; allograft), meniscal procedure (suture/resection), cartilage procedure (yes/no), and additional ligament procedures (medial collateral ligament, lateral collateral ligament, posterior cruciate ligament, posterolateral corner) at the time of primary ACLR. Furthermore, we examined possible trends in age at injury and age at ACLR, the percentage of additional meniscal procedures, and time from injury to primary ACLR dependent on whether an additional meniscal procedure was performed or not.

Our primary outcome was the incidence of primary ACLR according to age at the time of reconstruction, by sex, for each year in the time period 2005 to 2021. Secondary outcomes were (1) patient characteristics (age at injury and age at reconstruction, time from injury to surgery, and activity at injury); (2) surgical details (treatment of meniscal injuries [suture/resection], cartilage injuries, graft choice and graft survival, and number of the revision surgeries performed); and (3) patient-reported outcomes (KOOS preoperatively and at 2, 5, and 10 years after primary ACLR).

### Statistical Analysis

The incidence of ACLRs was calculated by comparing the number of patients registered in the NKLR with population data from the official website of the National Statistical Institute of Norway.^43^ We examined time trends using simple linear regression and calculated the moving 3-year average. We also prepared cumulative sum graphs of the deviations from the first 3-year average annual incidence (2005-2007) to detect smaller shifts over time.^54^ To describe patient characteristics and examine trends in surgical details, we arbitrarily divided the data set into 3 groups corresponding to time periods (see Table 3). The groups were compared using the Pearson chi-square test for categorical variables and the Kruskal-Wallis or Mann-Whitney test for nonparametric, continuous variables. A *P* value of <.05 was considered statistically significant, and all tests were 2-sided.

Patient characteristics and surgical details were presented as means and standard deviations for continuous variables and frequencies and percentages for categorical variables. The variable “activity at time of injury” was stratified according to age groups. Graft survival was analyzed using the Kaplan-Meier method, with revision surgery as the endpoint. The difference in survival between hamstring and bone–patellar tendon–bone (BPTB) grafts was assessed using the log-rank test. For patient-reported outcomes, means and standard deviations for each of the 5 KOOS subscales were calculated. The mean preoperative and 2-year KOOS values were compared to look for improvement after surgical intervention, and analyzed using paired *t* tests. Missing items in any KOOS subscale were treated according to protocol.^21^ The KOOS response rate was included because the follow-up duration was different for individual patients (Figure 1). To assess the potential impact of multiligament reconstructed patients on the KOOS results, a sensitivity analysis was conducted. Analyses and tests were performed using Stata SE 17 (StataCorp LLC), SPSS software Version 26.0 (IBM Corp), SPC for Excel (BPI Consulting), and R Version 4.1.2 (The R Project for Statistical Computing).^47^

### Ethics

NKLR was approved by the Norwegian Data Inspectorate in 2004. Inclusion was based on written informed consent signed by the patient or by the parent or caretaker if the patient was <16 years of age. The present study protocol was approved by the Norwegian Regional Committee for Medical and Health Research Ethics (No. 95244) and by the data protection officer at Akershus University Hospital.

## Results

Among the 29,264 primary ACLRs performed in Norway between 2005 and 2021, 1484 ACLRs in 1476 patients met our inclusion criteria. Among these, 947 were boys and 529 were girls (Figure 1). The mean follow-up time for the included patients was 8.1 years (range, 1-17 years).

**Figure 2.**
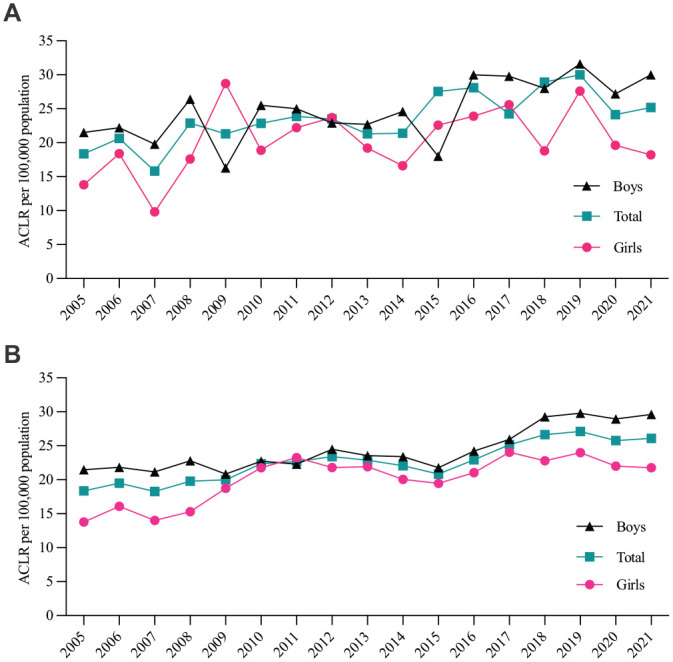
(A) Yearly incidence of pediatric anterior cruciate ligament reconstruction (ACLR) for boys (10-16 years), girls (10-14 years), and both sexes combined (total), per 100,000 population. (B) Three-year moving average of the incidence per 100,000 population, by sex.

The overall incidence of primary ACLR increased significantly (*r*^2^ = 0.44; *P* < .01) during the study period (Figure 2A). The moving 3-year average incidence increased steadily from 18 to 26 per 100,000 between the first and last 3-year periods (Figure 2B). When the average incidences of the first and last 3-year periods (2005-2007 vs 2019-2021) were compared, the relative increase in pediatric ACLR was 40% for boys and 55% for girls. This corresponds to an annual mean increase of 2.1% for boys and 2.8% for girls (see the Appendix, available in the online version of this article).

The mean age at the time of ACLR was 15.8 years for boys and 14.3 years for girls (Table 1). The age distribution was skewed, with 55% of the boys being 16 years of age and 77% of the girls being 14 years of age.

**Table 1:** Baseline Patient Characteristics^*a*^

Characteristic	Boys (n = 955 cases)	Girls (n = 529 cases)	Total (n = 1484 cases)
Age of participants, y, range	10-16	10-14	10-16
Age at injury, y	14.9 (1.7)	13.5 (1.3)	14.4 (1.6)
Age at ACLR, y	15.8 (1.0)	14.3 (0.6)	15.3 (1.1)
Height, cm	177 (8.3); n = 733	165 (5.8); n = 416	
Weight, kg	70.1 (12.1); n = 73	60.0 (9.3); n = 412	

a Values are presented as mean (SD) unless specified otherwise. If >4% missing data, n is specified. ACLR, anterior cruciate ligament reconstruction.

**Figure 3.**
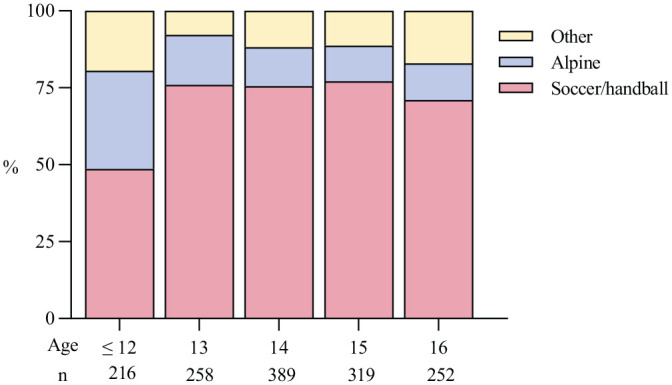
Activity at injury (in percentages), by age. Of all the injuries, 96.4% were sports-related. “Other” represents a range of other sports, such as basketball, floorball, and martial arts.

Sports activities were involved in almost all injuries that led to ACLR. Soccer and handball were the most common activities reported in the older age groups. Alpine sports, which include twin tip, freestyle, snowboard, and slopestyle, accounted for a larger proportion of injuries in those <12 years of age (Figure 3).

Concurrent meniscal procedures (meniscal sutures and resections) were performed in 66% of the cases (Table 2). Multiligament injuries that required surgical intervention were observed in 2.8% of the cases. The number of multiligament injuries did not increase over time, and a sensitivity analysis with the patients with multiligament reconstruction removed showed no effect on the mean KOOS subscores.

**Table 2:** Surgical Details^*a*^

	No. of Cases, n (%)
Graft	
BPTB	511 (34.4)
Hamstring	870 (58.6)
Quadriceps	89 (6.0)
Other	14 (0.9)
Additional procedures	
Meniscal suture only	542 (36.5)
Meniscal resection only	350 (23.6)
Combined suture/resection	84 (5.7)
Cartilage procedure	19 (1.3)
Multiligament injuries^*b*^	42 (2.8)
MCL	24 (1.6)
LCL	5 (0.3)
PCL	2 (0.1)
PLC	3 (0.2)
>2 ligaments reconstructed	8 (0.5)
ACL revision	149 (10.0)

a ACL, anterior cruciate ligament; BPTB, bone–patellar tendon–bone; LCL, lateral collateral ligament; MCL, medial collateral ligament; PCL, posterior cruciate ligament; PLC, posterolateral corner.

b Multiligament injuries: additional reconstructed ligaments at the time of the primary ACL reconstruction.

**Figure 4.**
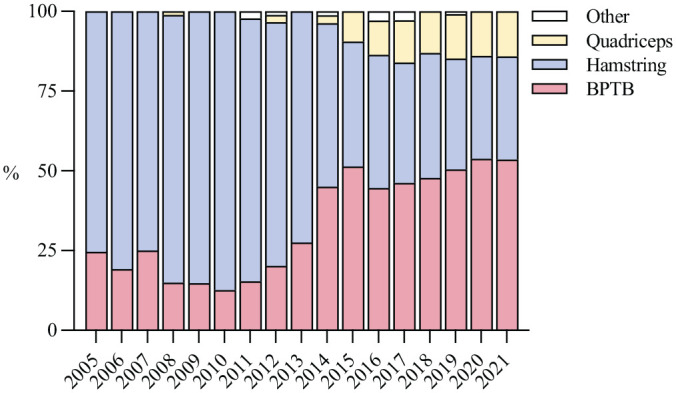
Choice of graft per year, in percentages. “Other” represents sutures and allografts. BPTB, bone–patellar tendon–bone.

The most frequent choice of ACL graft was the hamstring tendon autograft (59%), although the BPTB has become increasingly popular over the past 17 years (rising from 25% to 54%) (Figure 4).

The median time from injury to surgery was reduced from 6.5 to 5.4 months when comparing the first and last time period (*P* = .01) (Table 3). There were no significant differences in median time to surgery between patients who underwent a concurrent meniscal procedure and those who did not, in all 3 time periods (*P* = .54, *P* = .32, and *P* = .80, respectively) (Table 3). The overall proportion of patients who underwent a concurrent meniscal procedure at the time of the primary ACLR increased steadily over time, from 45% to 62% (*P* < .0001). There was a major shift in the meniscal procedures performed, from resection to suture (Table 3).

**Table 3:** Trends in Pediatric ACLR^*a*^

	2005-2010 (n = 462)	2011-2015 (n = 414)	2016-2021 (n = 608)
Sex, n			
Male	298	255	402
Female	164	159	206
Age at surgery, y, mean (SD)			
Boys	15.9 (1.0)	15.9 (0.9)	15.8 (1.1)
Girls	14.3 (0.6)	14.6 (0.6)	14.3 (0.6)
Time from injury to surgery, mo	6.5 (0.1-98.5)	6.7 (0.0-100.5)	5.4 (0.1-135.3)
With meniscal procedure^*b*^	6.6 (0.1-71.5); n = 216	7.0 (0.0-100.5); n = 223	5.7 (0.1-195.4); n = 366
Without meniscal procedure	6.4 (0.5-98.5); n = 233	6.1 (0.0-78.1); n = 176	5.3 (0.1-111.8); n = 220
ACLR with meniscal procedure, %	45.4	53.6	61.9
Suture, %	19.0	26.8	42.6
Resection, %	24.0	20.3	11.7
Combined, %	2.4	6.5	7.6

a Values are presented as median (range) unless specified otherwise. ACLR, anterior cruciate ligament reconstruction.

b Meniscal procedures include meniscal suture and/or resection.

**Figure 5.**
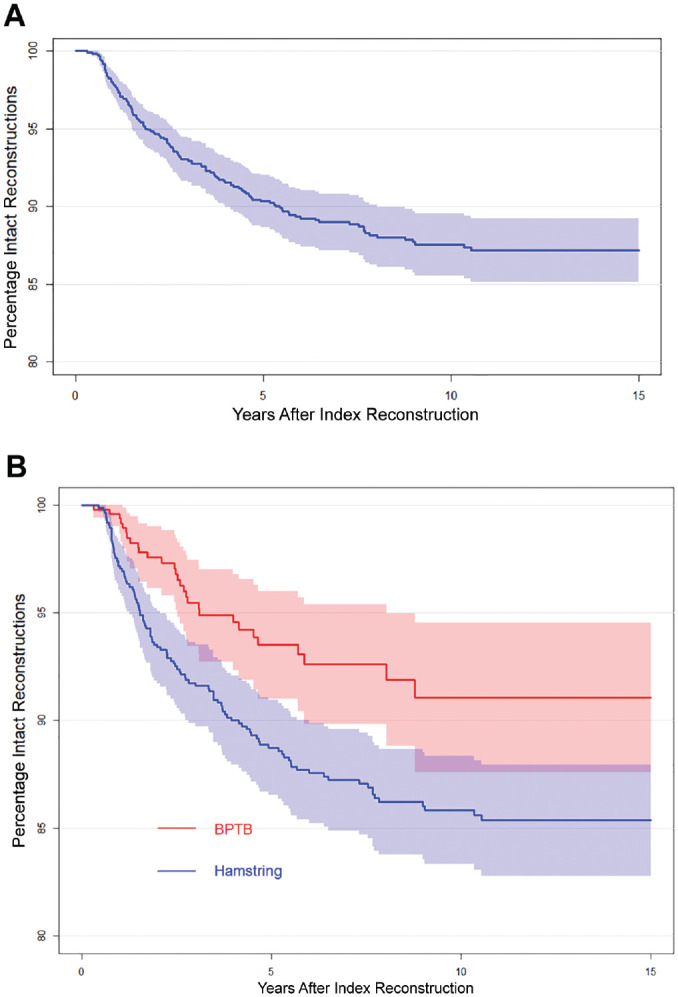
Kaplan-Meier survival curves for revision surgeries for (A) all patients (B) and those who received a bone–patellar tendon–bone autograft (BPTB) or hamstring tendon autograft.

A total of 149 cases (10.0%) underwent a revision ACLR. The mean time from the primary ACLR to revision was 2.9 years (95% CI, 2.5-3.3 years). The 5-year revision rate was 9.9% (95% CI, 8.2%-11.4%) (Figure 5A). The revision rate was higher for hamstring grafts than for BPTB, and the difference was statistically significant (*P* = .004) (Figure 5B). The mean ages at the time of revision was 15.1 years (SD, 1.2 years) for those who received a hamstring graft, and 15.8 (SD, 1.0 years) for those who received a patellar tendon graft.

**Figure 6.**
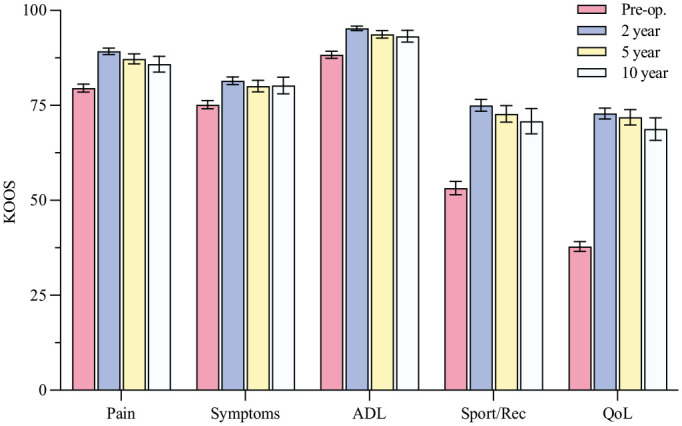
Knee injury and Osteoarthritis Outcome Score (KOOS) preoperatively (Pre-op) and at the 2-, 5-, and 10-year follow-up visits, with 95% CI. A score of 100 indicates no symptoms, and 0 indicates severe knee problems. For missing data, see the flowchart in Figure 1. ADL, Activities of Daily Living; QoL, Quality of Life; Sport/Rec, Sport and Recreation.

In terms of subjective patient-reported outcomes, the KOOS Sport/Rec and QoL subscales had the worst long-term outcomes, with mean values close to 70 (Figure 6). Significant improvements were observed between preoperative and 2-year KOOS values across all subscales (*P* < .001), with the largest improvement in the Sport/Rec and QoL subscales.

## Discussion

The main finding in this nationwide study of pediatric ACLR in Norway was that the incidence increased by 40% and 55% for boys and girls, respectively, during the study period. The activity at the time of injury varied by age group; alpine skiing was most common in children <12 years old, whereas soccer and handball dominated in older children. The time from injury to surgery decreased over time, and was independent of whether additional meniscal procedures were performed or not. The proportion of additional meniscal procedures at primary ACLR increased markedly from 45% to 62%. Long-term follow-up revealed a 10% revision rate, and mean KOOS values for Sport/Rec and QoL subscales ranging between 72 and 75 at 2, 5, and 10 years postoperatively.

The increased incidence of ACLR in our study was consistent with reports from the United States, Australia, and Finland.^7,42,52,55^ To our knowledge, only a single study has reported a decline in pediatric ACLR, a proposed result of implemented prevention programs.^7^ However, the finding relied solely on insurance data, which could be affected by coinciding changes in insurance agreements. Our more recent 2021 results showed a steady increase, in spite of spanning a time period that encompassed both increased awareness of prevention, and the beginning of the COVID-19 pandemic. Although we observed a small decrease in the incidence for 2020 and 2021 (Figure 2A), it was insufficient to affect the 3-year moving average (Figure 2B), and it was far from the 25% reduction observed in the NKLR for all ages in the same time period.^46^ It is worth noting that most previous studies have reported increasing incidences of ACL injury as well as ACLR.^42,52^ However, the true incidence of ACL injury is uncertain, mainly because of the lack of reliable data on patients treated nonoperatively. In our study, we assessed ACLRs only. By using a national data set with high quality and validity, we had reliable information on the majority of the performed ACLRs. However, the number of ACL injuries remains unknown, providing us with only a partial view of the total injury burden.

The Norwegian approach of primarily nonoperative treatment should be kept in mind when interpreting our findings. The patients who underwent ACLR in our study may represent a selected group of “noncopers,” that is, patients who do not cope well after ACL injury.^18,40^ This may affect some of the findings, such as the number of additional meniscal injuries and patient-reported outcomes, and may introduce selection bias if compared to a cohort of patients who underwent ACLR early. Taking into account the Norwegian treatment approach, the observed increase in ACLRs may imply an even larger increase in ACL injuries, which is worrisome.^11,30^

The cause of the increase in ACLR, and the possible increase in ACL injuries, is probably multifactorial. With 96% of the injuries being sports-related, the increase may be attributed to earlier sports specialization, with intense training programs and a higher level of competition at a younger age, potentially limiting free play and exposure to adversity. The increase in ACLR may also be a result of diagnostic improvements (magnetic resonance imaging) and public awareness of both the injury and the reported association with poorer outcomes in patients who did not undergo operation.^36^ It could also be explained by the advancement of techniques and equipment required for skeletally immature patients and the subsequently increased number of orthopaedic surgeons with experience in performing these surgical procedures.^45^ Furthermore, a change in surgical indication may have influenced the change; the increased focus on preserving the menisci in recent years may have led to a shift toward simultaneous ACLR if there is an additional repairable meniscal injury. Approximately 66% of the knees in our cohort presented with concurrent meniscal injuries that were surgically treated at the time of ACLR. This proportion is consistent with the report from the newly developed Paediatric ACL Monitoring Initiative^32^ but higher than in other studies.^33^ The focus on “saving the meniscus”^41^ is reflected in the present study in a shift from meniscal resection to repair, resulting in a statistically significant increase in meniscal sutures from 19% to 43% (Table 3).^2,41^ However, it is unclear whether the meniscal injury occurred at the time of ACL injury or as a consequence of persistent instability.

The timing of surgery is a debated topic.^33^ Proponents for early surgery argue that stabilizing the knee early with ACLR will reduce the risk of new meniscal and cartilage injuries. The association between delayed surgery and increased risk of meniscal injuries is supported by several studies.^1,22^ However, it is important to be aware that studies advocating early ACLR to protect the menisci and cartilage from further damage have methodological limitations and are prone to selection bias.^9,19,36^ In a cohort of 47 patients, Ekås et al^10^ reported that 16 patients (34%) sustained new meniscal injuries at a mean follow-up of 9.5 years, following the Norwegian treatment approach described earlier. In our study, the time from injury to surgery was reduced, but the rate of meniscal repairs increased. This might reflect a general trend toward repairing the menisci at initial presentation rather than secondary meniscal injury due to delayed ACLR. Preserving the menisci to optimize future knee health is a favorable and encouraging trend. At the same time, the apparent increase in meniscal procedures is worrisome because it may also indicate an increased degree of severity of the knee injuries leading to surgery.

At follow-up, the mean scores on the KOOS Sport/Rec and QoL subscales were lower compared to a population with healthy knees but higher than those observed in the adult population in the NKLR.^27,46^ Our study revealed scores around 70 for patients who underwent reconstruction, indicating a 30% reduction in self-reported knee function at long-term follow-up. This finding highlights the impact and severity of ACL injury, even after surgical reconstruction. Several surgical techniques have been described in the literature on pediatric ACLR. However, there is no consensus on which technique is superior, and detailed information on the technique used is not available in the NKLR. Irrespective of the technique used, it is clear that the outcome after surgical treatment is still not ideal, even though a statistically and clinically significant improvement was observed between preoperative and postoperative KOOS values. A previous study using an initial active rehabilitation treatment approach in 44 children with a mean 8-year follow-up showed mean KOOS values of 87 and 84 for the Sport/Rec and QoL subscales, respectively, in patients who did not undergo operation and, correspondingly, 87 and 80 in those who underwent ACLR.^11^ This may indicate that “copers,” that is, those who compensate well after an ACL injury, do exist in this population and that surgery may not necessarily be the optimal treatment for all.

According to the literature, pediatric patients have the highest reported risk of recurrent injury of all age groups.^2^ In our study, approximately 10% had undergone a revision ACLR within 5 years after primary ACLR (Figure 5A). This revision rate is higher compared to the adult population, with a rate of 4.2% to 4.8%.^35,46^ However, it is similar to rates seen in other pediatric populations, such as in those <19 years old who return to pivoting sports, where revision rates of 13% to 15% have been reported.^20^ Even if these patients return to sport, the alarmingly high risk of a new injury that leads to revision at an early age will have implications for their future knee-related health, especially with regard to knee osteoarthritis.^23^

We found a higher revision rate for patients with hamstring grafts than for those with patellar tendon grafts in our study (Figure 5B). It is possible that patient characteristics could account for this discrepancy. For example, younger skeletally immature patients who are at higher risk of revision may be more likely to receive a soft tissue graft, which is represented in the lower mean age for the patients who received a hamstring graft. However, our findings align with those reported in studies involving adults^35^ and may be attributed to inherent factors of the 2 graft types. In addition, it is worth noting that not all graft failures were necessarily recorded, as only revision surgeries are mandatorily reported in the NKLR. Therefore, the rate of ACL graft failures might be higher, emphasizing the severity and burden of an ACL injury even when reconstructed.

Our study revealed a shift towards increased use of BTPB over time, in line with the trend shown in adults.^46^ As grafts with bone blocks are not advised in skeletally immature patients, the majority of the increase of BPTB usage is likely to reflect patients who have just reached skeletal maturity. This leads to a final note on nomenclature. The term “pediatric” is widely used but lacks a clear definition in the literature concerning ACLR. Age cutoffs are commonly used as a proxy for the lack of concrete radiological data on skeletal maturity. This also applies to our study, where the patients operated on with BPTB grafts were likely to represent skeletally mature individuals.^2^ Nevertheless, these young patients face different challenges than adults do, and are a population at definite risk. They therefore deserve our fullest attention—regardless of the exact status of their skeletal maturity.^5,51,53^

### Limitations

In general, cohort and register studies may suffer from recruitment bias and loss to follow-up in patient-reported data. Yet, we believe the NKLR data did allow us to accurately describe pediatric ACLR in Norway for the past 17 years because of its high compliance and coverage for primary reconstructions. The response rate for patient-reported outcomes at the 2-year follow-up in the present study was higher than the general response rate to the NKLR (67% vs 60%).^46^ Although this response rate is considered high for a prospective cohort in a national register setting, we acknowledge that respondents may differ from nonrespondents. However, data from a comparable Scandinavian register found only minor nonclinically relevant differences in KOOS between respondents and nonrespondents.^37^

In addition, some studies have questioned the utility of KOOS in evaluating patients who undergo ACLR, especially in pediatric patients.^17,26,34^ It is important that the patient-reported outcome measures used actually measure what is intended. Nevertheless, the KOOS is validated for the adult population, and the large majority of our patients had reached adulthood by the time of their KOOS reply at 5- and 10-year follow-up. Finally, it should be noted that during the study period, the register did not fully capture the reconstruction technique used (anatomic vs nonanatomic) or potential additional extra-articular procedures, precluding us from exploring their effect on revision rates and KOOS values.

### Implications

Our study provides a comprehensive understanding of clinical practice over 17 years in a large, longitudinal cohort. The results are in line with the rising incidence of pediatric ACLR reported internationally, adding valid nationwide data to the discussion on management and hopefully leading to increased awareness and focus on this severe injury in the youngest.

Our finding that the activity at the time of injury varies for different age groups has important implications for injury prevention. Identifying sports at risk is helpful for targeting exercise-based prevention programs, which have been proven effective.^39,44,48^ Our patient-reported outcomes confirm that ACL injury followed by ACLR imposes a serious long-term health burden in this young and otherwise healthy population. This is important when counseling the patients and their families about the injury’s prognosis. Reinjury risk with a possible need for revision surgery has to be conveyed, and the desire to return to pivoting sports should be balanced with the risk of reinjury and with the future knee health in mind—even after ACLR.

Our study also serves as a baseline for future research and highlights that the outcomes after pediatric ACLR are still not ideal. We need to increase the knowledge on the total ACL injury burden, including data from patients treated nonoperatively. Research should focus on optimizing treatment algorithms to find the optimal surgical technique when reconstruction is performed, to improve the short- and long-term outcomes.

## Conclusion

The incidence of pediatric ACLRs in Norway increased from 18 to 26 per 100,000 population, corresponding to an increase of 40% for boys and 55% for girls from 2005 to 2021. In children <12 years old, alpine skiing was the most common activity at injury, while soccer and handball were most common for those >12 years old. The percentage of additional meniscal procedures at primary ACLR increased from 45% to 62% during the study period, whereas time from injury to surgery decreased. The 5-year revision rate was 9.9%. The 5- and 10-year mean KOOS values of QoL and Sport/Rec ranged between 72 and 75, indicating a long-lasting and markedly reduced knee function. ACL injuries and reconstructions impose an increasing health burden in the young population, with long-term knee health deficits that warrant further attention.

## Supplemental Material

sj-pdf-1-ajs-10.1177_03635465231185742 – Supplemental material for Major Increase in Incidence of Pediatric ACL Reconstructions From 2005 to 2021: A Study From the Norwegian Knee Ligament Register Click here for additional data file. Supplemental material, sj-pdf-1-ajs-10.1177_03635465231185742 for Major Increase in Incidence of Pediatric ACL Reconstructions From 2005 to 2021: A Study From the Norwegian Knee Ligament Register by Caroline E. v. W. Kooy, Rune B. Jakobsen, Anne M. Fenstad, Andreas Persson, Håvard Visnes, Lars Engebretsen and Guri R. Ekås in The American Journal of Sports Medicine
