# Supplementary material for: Major Increase in Incidence of Pediatric ACL Reconstructions From 2005 to 2021: A Study From the Norwegian Knee Ligament Register
Source: Am J Sports Med. 2023 Jul 27;51(11):2891–9. doi: 10.1177/03635465231185742 (PMC10478322; doi:10.1177/03635465231185742)
Supplement: sj-pdf-1-ajs-10.1177_03635465231185742 – Supplemental material for Major Increase in Incidence of Pediatric ACL Reconstructions From 2005 to 2021: A Study From the Norwegian Knee Ligament Register [file sj-pdf-1-ajs-10.1177_03635465231185742.pdf]

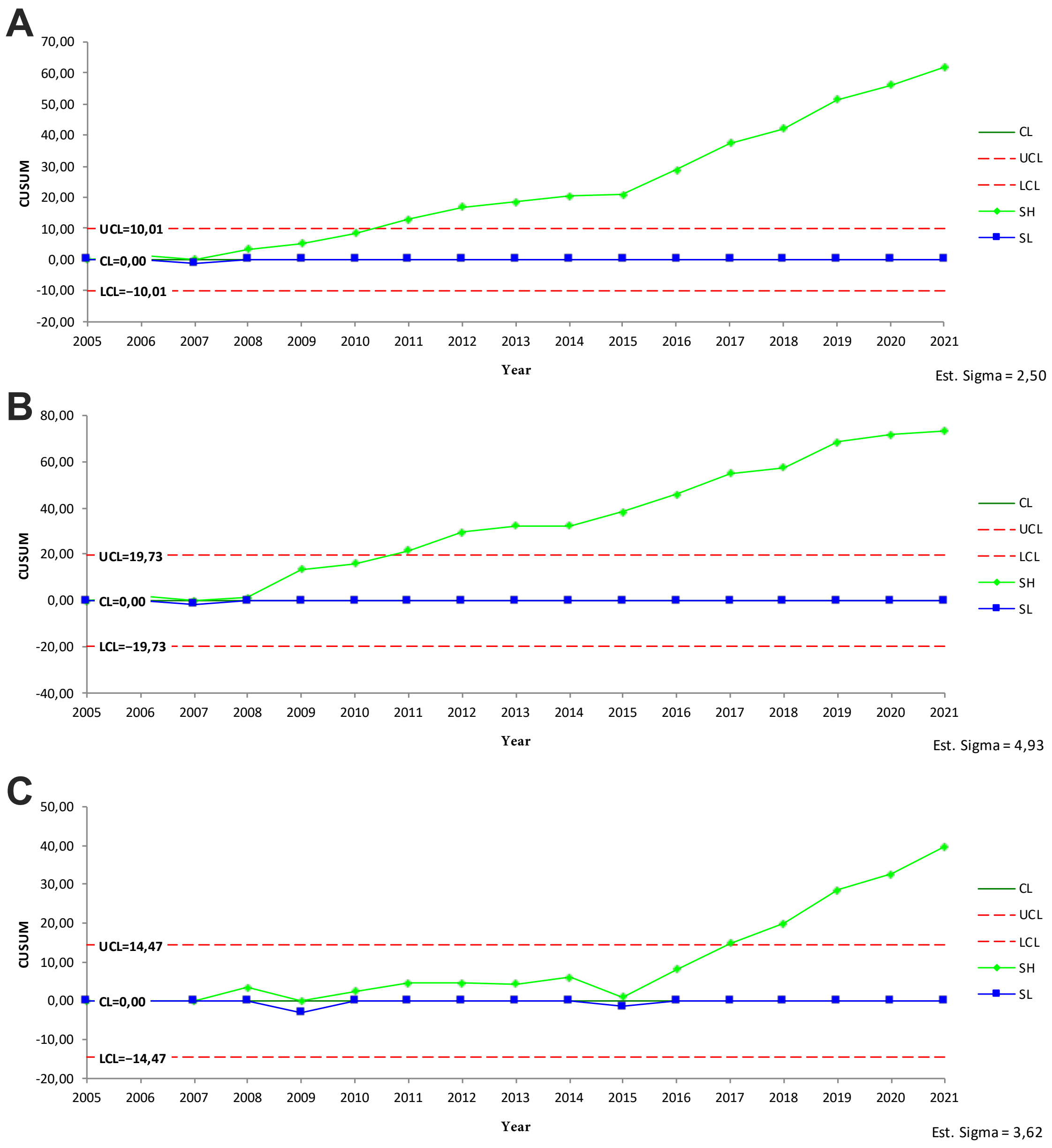

Appendix figure 1: CUSUM-plots

Cumulated sum (CUSUM) of deviations from the first three-year average incidence plotted as time series year by year with control lines showing a marked increase for both sexes combined (panel A), females (panel B) and males (panel C). CL (centerline), UCL/LCL (upper/lower control limit), SH (cumulative sum on the high side of the first three-year average), SL (cumulative sum on the low side of the first three-year average).
